# Genome-wide distribution and putative regulatory roles of G-quadruplex motifs in oral bacteria

**DOI:** 10.3389/fgene.2026.1961845

**Published:** 2026-10-29

**Authors:** Yizhou Wang, Zongyun Fu, Pengcheng Gao, Yaozhong Chen

**Affiliations:** Department of Operative Dentistry and Endodontics, Zhongda Hospital, Medical College, Southeast University, Nanjing, China

**Keywords:** comparative genomics, evolution, G-quadraplexes, oral bacteria, regulation

## Abstract

**Introduction:**

G-quadruplexes (G4s) are non-canonical DNA secondary structures with established regulatory roles in eukaryotes, yet their prevalence, distribution, and functional significance in bacteria remain poorly understood.

**Methods:**

We conducted a comprehensive survey of G4 motifs across 381 reference genomes representing 150 genera of oral bacteria. Computational prediction was used to identify G4 motifs and characterize their genomic distribution, GC-content dependence, and functional associations. Additionally, comparative analysis of orthologous promoters across 35 genera was performed to investigate the evolutionary conservation and turnover of promoter-associated G4 motifs.

**Results:**

We identified 48,234 G4 motifs and found that their genomic density increased exponentially with GC content. G4 motifs were enriched in promoter regions relative to genome-wide expectations and preferentially associated with genes involved in signal transduction, virulence, transcriptional regulation, and transport. Comparative analysis revealed that promoter-associated G4s were shared among congeneric species more frequently than expected by chance, but their sharing declined sharply with increasing phylogenetic distance. Moreover, G4-forming sequences showed no greater conservation than other G-rich sequences within the same promoters, suggesting rapid evolutionary turnover.

**Discussion:**

These findings demonstrate that G4 motifs are non-randomly distributed across oral bacterial genomes and exhibit evolutionary patterns consistent with inheritance and rapid turnover. The enrichment of G4s in promoters of functionally relevant genes highlights candidate loci for future experimental investigation of G4-mediated transcriptional regulation in oral bacteria.

## 1 Introduction

G-quadruplexes (G4s) are non-canonical nucleic acid secondary structures formed by guanine-rich sequences, in which four guanine bases associate through Hoogsteen hydrogen bonding to form planar G-quartets that stack upon one another to create a thermodynamically stable four-stranded structure (Burge et al., 2006; Davis, 2004). The canonical G4-forming motif consists of four runs of at least three consecutive guanines separated by loops of variable length, giving rise to diverse topological conformations including parallel, antiparallel, and hybrid structures (Spiegel et al., 2020). The stability of G4 structures is enhanced by monovalent cations, particularly potassium, which coordinates between stacked G-quartets (Bhattacharyya et al., 2016).

In eukaryotes, G4s have been extensively characterized as regulatory elements with diverse biological functions. Over 700,000 potential G4-forming sequences have been identified in the human genome, with significant enrichment in gene promoters, 5′untranslated regions, and telomeric sequences (Huppert and Balasubramanian, 2005; Huppert and Balasubramanian, 2007). Functional studies have established roles for G4s in transcription regulation, where they can act as both activators and repressors depending on their position and orientation relative to the transcription start site (Varshney et al., 2020). G4 structures at replication origins contribute to origin firing (Valton and Prioleau, 2016), while telomeric G4s are critical for chromosome end protection (Rhodes and Lipps, 2015). The biological importance of G4s is underscored by the existence of dedicated helicases, including RecQ family members and FANCJ, that resolve G4 structures during DNA replication and transcription (Mendoza et al., 2016; Brosh, 2013).

In contrast to the wealth of knowledge in eukaryotes, the understanding of G4 biology in bacteria is comparatively nascent (Fu et al., 2025a). Alternative nucleic acid structures, including G4s, are increasingly recognized as functional elements of microbial genomes that influence gene regulation, genome stability, stress adaptation and pathogenicity, and as potential antimicrobial targets (Yousuf and Bhat, 2026). Computational surveys have identified potential G4-forming sequences in several bacterial genomes, including *E. coli*, *Mycobacterium tuberculosis* (Perrone et al., 2017), *Deinococcus radiodurans* (Kota et al., 2015), and *Neisseria gonorrhoeae* (Cahoon and Seifert, 2009). In *Escherichia coli*, G4 motifs are enriched in regulatory regions and their presence in promoters can modulate gene expression *in vitro* and *in vivo* (Holder and Hartig, 2014; Kaplan et al., 2016). In mycobacteria, G4 structures have been implicated in the regulation of virulence-associated genes (Perrone et al., 2017). A particularly intriguing connection exists between G4 structures and phase variation in *Neisseria* species, where G4-forming sequences within pilin gene loci facilitate antigenic variation through recombination (Cahoon and Seifert, 2013). Despite these tantalizing observations, fundamental questions remain unresolved: whether bacterial G4s are merely passive byproducts of nucleotide composition or instead exhibit signatures of functional selection; whether they show consistent positional preferences relative to genes across diverse taxa; and how rapidly they evolve.

The human oral microbiome presents an ideal model system for addressing these questions at a community-wide scale. The oral cavity is one of the most diverse microbial habitats in the human body, harboring approximately 700 bacterial species organized into complex biofilm communities (Dewhirst et al., 2010; Chen et al., 2010). Oral bacteria span an enormous range of GC content (23%–75%), genome sizes (0.8–9.5 Mb), and ecological strategies, from obligate anaerobes in subgingival plaque to aerotolerant species on the tongue dorsum (Lamont et al., 2018). This natural variation in GC content is critically important for our analytical approach: because G4 formation requires clustered guanine tracts, G4 occurrence is expected to increase exponentially with GC content purely by chance. The wide GC range across oral bacteria provides the statistical leverage needed to distinguish G4 occurrence explained by nucleotide composition from deviations beyond compositional expectation. Furthermore, the oral microbiome includes both well-characterized pathogens (*Porphyromonas gingivalis*, *Streptococcus mutans*, *Aggregatibacter actinomycetemcomitans*) and commensals, enabling investigation of potential links between G4 distribution and pathogenic lifestyle (Hajishengallis and Lamont, 2012).

Here, we present the first pan-microbiome G4 atlas, comprehensively surveying G-quadruplex motifs across 381 reference genomes representing 150 genera of oral bacteria. We employ a computational pipeline (Molder et al., 2021) integrating G4 prediction with genomic context annotation, functional enrichment analysis, comparison with composition-preserving randomized genomes, and comparison of orthologous promoters among congeneric species. Our results reveal that G4 motifs in oral bacteria are non-randomly distributed, positionally enriched in promoter regions, over-represented relative to their sequence composition in a small subset of genomes, and preferentially associated with virulence and environmental response genes.

## 2 Results

### 2.1 G4 motifs are widespread but unevenly distributed across oral bacterial genomes

We applied g4predict (Parker, 2026) to 381 NCBI RefSeq reference genomes of oral bacteria, requiring a minimum of three G-quartet stacks and a maximum loop length of seven nucleotides for G4 prediction. This analysis identified a total of 48,234 G4 motifs across the dataset (Supplementary Table S1). The distribution was highly uneven across genomes: 63 genomes (16.5%) contained no predicted G4 motifs, while the most G4-rich genome, *Actinomyces dentalis*, harbored 2,210 motifs (Figure 1D). Among genomes with at least one G4, the median count was 37 and the mean was 151.7, reflecting a heavily right-skewed distribution. G4 density ranged from 0.0003 to 0.63 G4s per kilobase, spanning four orders of magnitude.

**FIGURE 1:**
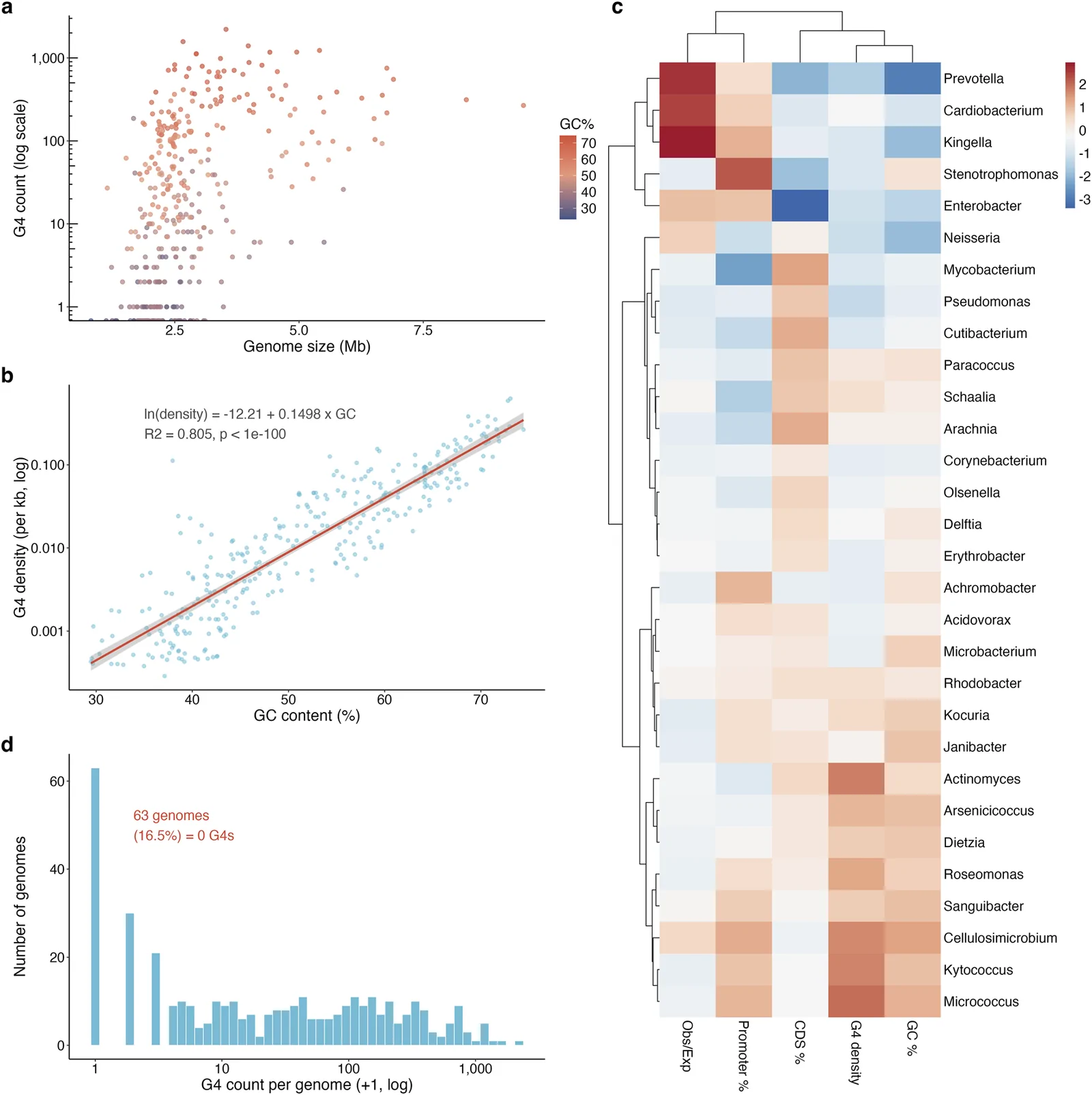
G-quadruplex distribution landscape across 381 oral bacterial genomes. **(a)** Scatter plot of genome size versus G4 count for each genome, colored by GC content, showing that GC content rather than genome size is the primary determinant of G4 abundance. **(b)** Exponential relationship between GC content and G4 density (per kilobase), with fitted regression line (ln (density) = −12.21 + 0.15 × GC%; R^2^ = 0.805, p < 10^−100^). Grey band indicates the 95% confidence interval of the fit. **(c)** Hierarchical clustering heatmap of z-scored G4 features across the top 30 genera by total G4 count. Features include G4 density, observed/expected ratio (relative to dinucleotide-preserving randomized genomes), percentage of G4s in promoter and CDS regions, and mean GC content. Ward’s minimum variance method was used for clustering. **(d)** Histogram of G4 counts per genome (log scale), highlighting that 63 genomes (16.5%) contain zero predicted G4 motifs.

The 381 genomes encompassed 150 genera across a broad phylogenetic range. The dataset was dominated by single-species genera: 83 genera (55.3%) were represented by a single species, 24 (16.0%) by two species, and 43 (28.7%) by three or more species (Supplementary Figure S4c). The most species-rich genera were *Corynebacterium* (27 species), *Streptococcus*, *Neisseria*, *Prevotella*, and *Actinomyces*. Genome sizes ranged from 0.81 Mb (*Treponema denticola*) to 9.52 Mb (*Bradyrhizobium oligotrophicum*), with a mean of 2.77 Mb and median of 2.32 Mb (Supplementary Figure S4b). GC content exhibited a bimodal distribution (Supplementary Figure S4a), with peaks at approximately 35%–40% GC (dominated by Firmicutes and Bacteroidetes) and 60%–70% GC (dominated by Actinobacteria and Proteobacteria). All 63 genomes lacking G4 motifs had GC content below 45%, coinciding with the low-GC peak of this distribution.

The relationship between G4 density and GC content was strongly exponential (ln (density) = −12.21 + 0.15 × GC%; R^2^ = 0.805, p < 10^−100^; Figure 1B), confirming that nucleotide composition is the primary determinant of G4 occurrence (Fu et al., 2025a). This exponential scaling is consistent with theoretical expectations, as the probability of finding four G-runs of three or more consecutive guanines within a defined window increases approximately as (GC/2)^12^ under a random nucleotide model. Notably, all 63 genomes lacking G4 motifs had GC content below 45%, and no genome with GC content above 50% was devoid of G4s (Figure 1a). GC content alone explained 80.5% of the variance in log G4 density, and the remaining variation was structured rather than random: adding phylum explained a further 3.3% (F-test p = 3.0 × 10^−10^), whereas genome size added only 0.3% (p = 0.025) (Supplementary Figure S5). At the same GC content, Campylobacterota and Bacteroidota genomes carried more G4 motifs than the fit predicted and Bacillota genomes fewer (Supplementary Figures S5b,c); Section 2.3 examines these deviations against explicit null models.

Genome size showed a weaker and more nuanced association with G4 count (Figure 1a). Low-GC genomes consistently had few or no G4s regardless of genome size, while high-GC genomes showed positive scaling of G4 count with genome size, as expected from the larger mutational target. The ten genera with the highest total G4 counts were *Actinomyces* (10,931 G4s across 12 species), *Corynebacterium* (5,423 across 27 species), *Roseomonas* (2,406 across 2 species), *Schaalia* (1,543 across 4 species), and *Neisseria* (1,453 across 15 species), all with mean GC content exceeding 50%.

Hierarchical clustering of genus-level G4 features revealed distinct taxonomic patterns (Figure 1c). High-GC genera such as *Corynebacterium*, *Micrococcus*, and *Kytococcus* clustered together, characterized by high G4 density and elevated CDS G4 fractions. A separate cluster of moderate-GC genera, including *Prevotella*, *Cardiobacterium*, *Kingella* and *Neisseria*, showed high observed-to-expected ratios despite moderate G4 densities, indicating more G4 motifs than expected from their sequence composition (Section 2.3). An extended heatmap of the top 50 genera, including row annotations for GC content, species count, and total G4 count, is provided in Supplementary Figure S3.

### 2.2 G4s are enriched in promoter regions and associated with regulatory and virulence genes

To characterize the genomic context of G4 motifs, we defined five genomic region categories (promoter, rRNA, tRNA, CDS, intergenic) and assigned each G4 to exactly one region using a priority-based hierarchy (promoter > rRNA > tRNA > CDS > intergenic) to prevent double-counting of G4s overlapping multiple features. Promoter regions were defined as the 300 bp upstream of each annotated coding sequence start. Of the 48,234 G4 motifs, 17,284 (35.8%) were assigned to promoter regions, 27,152 (56.3%) to coding sequences, and 3,798 (7.9%) to intergenic regions (Figure 2a). No G4s were assigned to rRNA or tRNA regions after priority-based filtering.

**FIGURE 2:**
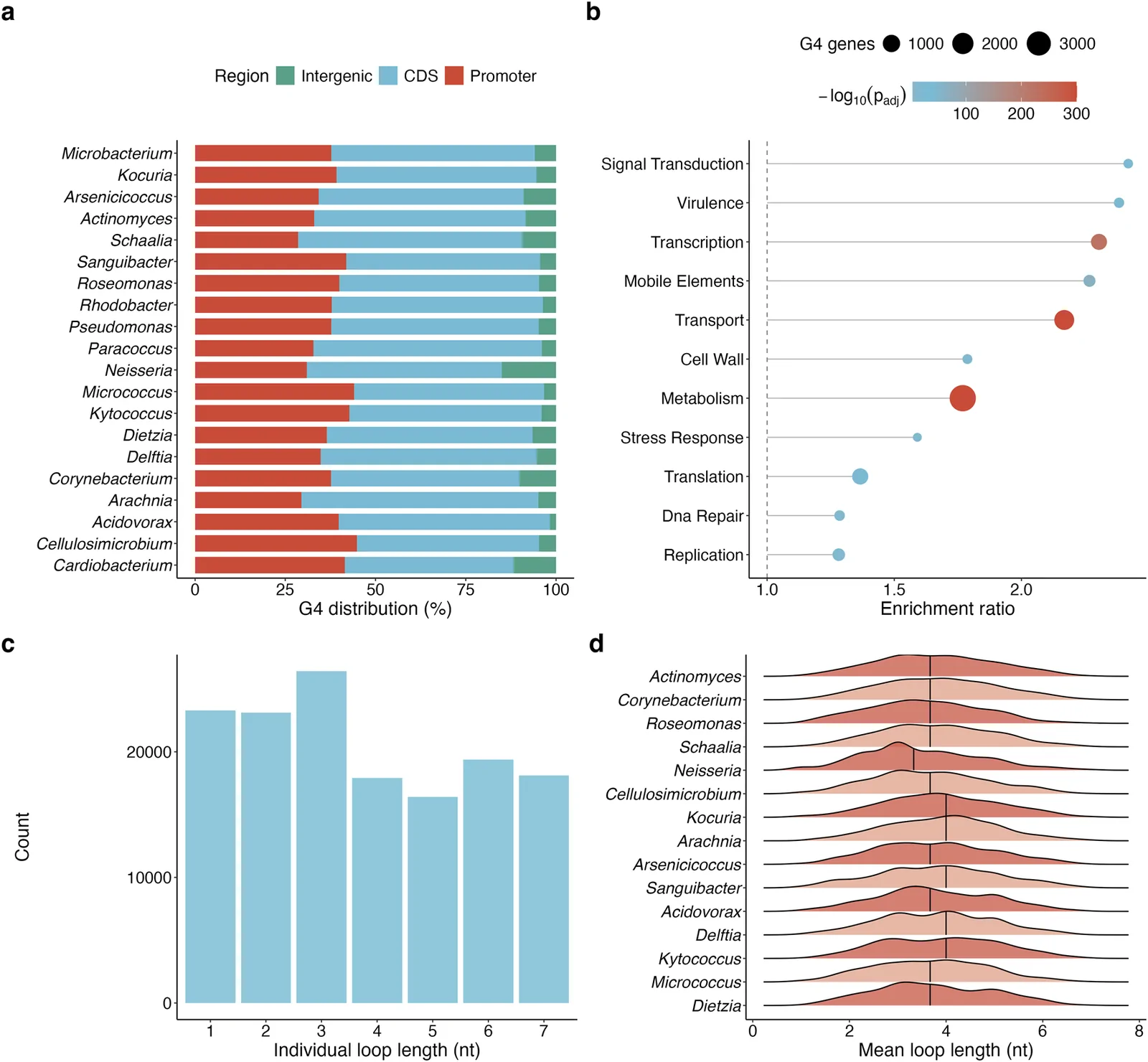
Genomic context and functional associations of G4 motifs. **(a)** Stacked bar chart showing the distribution of G4 motifs across genomic regions (promoter, CDS, intergenic) for the top 20 genera by total G4 count. Genera are ordered by promoter fraction. **(b)** Functional enrichment analysis of genes with promoter G4 motifs using Fisher’s exact test with FDR correction. Dot size reflects the number of G4 genes in each functional category; color represents statistical significance (–log_10_ adjusted p-value). The vertical dashed line marks enrichment ratio = 1 (no enrichment). **(c)** Distribution of individual loop lengths (1–7 nucleotides) across all 48,234 G4 motifs. **(d)** Ridgeline density plots of mean loop length per G4 motif for the top 15 genera by G4 count. Vertical lines indicate medians. The consistent peak positions across genera indicate conserved structural preferences.

The 35.8% promoter assignment is notable because promoter regions (300 bp upstream of each CDS) comprise only approximately 10%–15% of the average bacterial genome, indicating a 2.5–3-fold enrichment of G4 motifs in promoter regions relative to random expectation. This promoter enrichment was remarkably consistent across genera: 18 of the top 20 genera by total G4 count showed promoter G4 fractions exceeding 25%, with *Microbacterium* (54%), *Kocuria* (49%), and *Arsenicicoccus* (45%) showing the highest promoter fractions (Figure 2a). The consistency of this pattern across phylogenetically diverse genera indicates that G4 motifs are non-randomly positioned relative to gene starts, although this positional association does not by itself show that they regulate transcription.

Functional enrichment analysis using Fisher’s exact test with Benjamini–Hochberg FDR correction revealed significant enrichment across all 11 functional categories tested, but with markedly different effect sizes (Figure 2b). The strongest enrichments were observed for signal transduction genes (2.4-fold, p_adj = 1.0 × 10^−38^), virulence factors (2.4-fold, p_adj = 3.0 × 10^−42^), transcription regulators (2.3-fold, p_adj = 4.1 × 10^−226^), mobile genetic elements (2.3-fold, p_adj = 6.1 × 10^−64^), and transport systems (2.2-fold, p_adj <10^−300^). In contrast, housekeeping functions including translation (1.4-fold), replication (1.3-fold), and DNA repair (1.3-fold) showed comparatively modest enrichment (Supplementary Table S3). This hierarchy of enrichment, strongest for regulatory and environmental response genes and weakest for core housekeeping functions, shows that promoter G4 motifs are preferentially associated with particular functional classes of genes. These are genomic associations; whether G4 structures at these promoters influence expression of the downstream genes remains to be tested experimentally.

Analysis of G4 structural features revealed a predominance of short loops (1–3 nucleotides), with 3-nucleotide loops being the most common, accounting for the highest frequency among all loop lengths (Fu et al., 2025b) (Figure 2c). All predicted G4 motifs contained exactly three G-quartet stacks (the minimum specified in our prediction parameters), with four G-runs of three consecutive guanines each. The motif length distribution was approximately normal, centered at a median of 23 nucleotides (range: 15–33 nt; Supplementary Figure S2a). Loop symmetry analysis revealed that only 5.0% of G4 motifs had all three loops of equal length (symmetric configuration), while the remaining 95.0% were asymmetric (Supplementary Figure S2b). The most frequent loop pattern was 3|3|3 (symmetric, 1,003 motifs), followed by 1|3|1 (394) and 1|3|3 (380) (Supplementary Figure S2d). Notably, mean loop length showed no systematic dependence on genomic GC content across genera (Supplementary Figure S2c), and the ridgeline distributions of loop lengths were consistent across the top 15 genera (Figure 2d), indicating that structural constraints on G4 motifs operate independently of overall nucleotide composition.

Strand bias analysis examined the distribution of G4 motifs between the template and non-template strands relative to the gene whose promoter or coding sequence contains the motif (Supplementary Figure S1). Across genera, the mean template strand fraction was 0.50 (Supplementary Figure S1a), although 59% of all genic G4 motifs lay on the template strand because several large high-GC genera, such as *Actinomyces* and *Corynebacterium*, were template-biased. However, 54 of 106 genera with at least 10 genic G4 motifs showed significant strand bias (FDR <0.05, binomial test), 39 toward the template strand and 15 toward the non-template strand. Most notably, *Eikenella* and *Kingella* showed strong template-strand bias (87.0% and 79.7% of G4s on the template strand, both p < 0.0001; Supplementary Figures S1a,b). Template-strand G4 motifs could impede transcription by blocking RNA polymerase progression, suggesting a potential role in transcriptional regulation or gene silencing. *Bartonella* showed the strongest non-template bias (98.4% non-template, p < 0.0001), where G4 formation during transcription could serve as a pause signal. The template strand fraction increased with GC content (Spearman ρ = 0.45, p < 0.0001; Supplementary Figure S1c), suggesting that genome composition, together with species-specific factors, shapes strand-asymmetric G4 distribution.

### 2.3 A subset of genomes contains more G4 motifs than expected from nucleotide composition

To assess whether G4 motifs occur more or less often than expected from nucleotide composition alone, we computed observed-to-expected (obs/exp) ratios for each genome. The expected number of G4 motifs was obtained by predicting G4 motifs, with the same settings, in randomized versions of each genome that preserve its dinucleotide composition, and observed and expected counts were compared with one-sided Poisson tests and Benjamini–Hochberg correction (Methods; Supplementary Methods S1). A simple analytical expectation, E (G4) = L × (GC/2)^12^, was not used because it underestimates expected counts about three-fold in low-GC genomes and overestimates them 1.7-fold in high-GC genomes (Supplementary Figure S6a). Genomes were classified into five categories based on their obs/exp ratios: strongly depleted (<0.3), depleted (0.3–0.7), expected (0.7–1.5), enriched (1.5–3.0), and strongly enriched (>3.0) (Supplementary Table S5).

Among the 318 genomes with at least one G4 motif, the obs/exp distribution was highly variable: 22 genomes (6.9%) were strongly depleted (obs/exp <0.3), 76 (23.9%) were depleted, 141 (44.3%) were within the expected range, 53 (16.7%) were enriched, and 26 (8.2%) were strongly enriched (obs/exp >3.0); 83 genomes were significantly enriched and 93 significantly depleted (FDR <0.05). The overall distribution was strongly right-skewed (median = 0.97, mean = 1.77), driven by a few outliers in the low-GC range. The obs/exp analysis showed no significant trend with GC content (Spearman ρ = −0.07, p = 0.22; Figures 3a,b), in contrast to the steep inverse relationship produced by the analytical expectation (Supplementary Figure S6a). Genomes in the lowest GC quartile (n = 80, mean GC = 37.5%) showed slightly higher obs/exp ratios (mean = 3.4, median = 1.2), while genomes in the highest GC quartile (n = 80, mean GC = 66.8%) were close to expectation (mean = 1.0, median = 0.96). The mid-low (mean GC = 45.8%) and mid-high (mean GC = 56.4%) quartiles showed intermediate values (mean obs/exp = 1.4 and 1.3, respectively). The difference between the lowest and highest quartiles was not significant (Mann–Whitney U = 3,664, p = 0.11). Box-and-jitter plots stratified by GC content bins showed no consistent gradient (Figure 3B): the median obs/exp was 2.0 in the 16 genomes below 35% GC and between 0.85 and 1.22 in all other bins.

**FIGURE 3:**
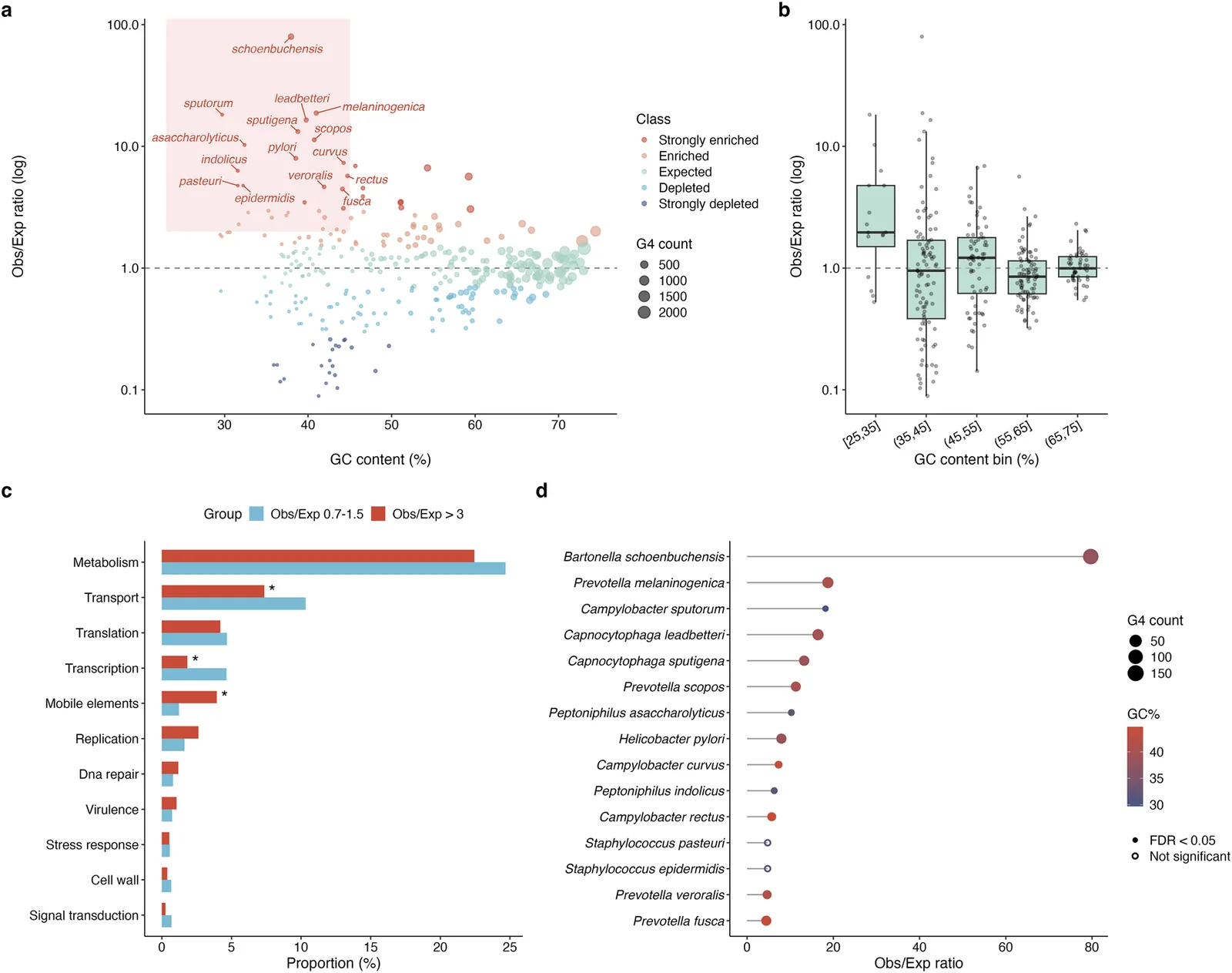
G4 enrichment relative to composition-preserving randomized genomes. **(a)** Scatter plot of GC content versus observed/expected (obs/exp) G4 ratio (log scale) for 318 genomes with at least one G4 motif, with expected counts from dinucleotide-preserving randomized genomes. Points are colored by obs/exp classification and sized by total G4 count. The red shaded zone highlights high-enrichment genomes (GC < 45%, obs/exp >2). Key species are labeled. The horizontal dashed line marks obs/exp = 1 (random expectation). **(b)** Box-and-jitter plots of obs/exp ratios stratified by GC content bins, showing no consistent trend with GC content (Mann–Whitney p = 0.11 between lowest and highest quartiles). **(c)** Comparison of functional profiles between high obs/exp genomes (>3) and expected genomes (0.7–1.5), showing proportional enrichment of mobile elements (3.2-fold) in outlier genomes; asterisks mark categories differing at FDR <0.05. **(d)** Lollipop chart of the top 15 high-enrichment genomes (obs/exp >2, GC < 45%), ranked by obs/exp ratio. Point size indicates G4 count; color indicates GC content; open symbols are not significant (FDR ≥0.05).

Several factors may contribute to this pattern. In high-GC genomes (>60%), guanine-rich sequences are abundant, and G4-forming motifs arise frequently as a passive consequence of nucleotide composition. Indeed, many high-GC genomes show obs/exp ratios below 1, indicating fewer G4s than expected by chance, possibly reflecting selection against G4 formation at sites where it would impede replication or transcription (Yadav et al., 2007), although this depletion was much weaker with randomized genomes than with the analytical expectation (Supplementary Figure S6a). In low-GC genomes (<40%), by contrast, G-runs are inherently rare, so the expected number of motifs under the null model is very small and obs/exp ratios become highly sensitive to small differences in the observed count. The high ratios in low-GC genomes are compatible with selective retention of G4-forming sequences, but they could also reflect local variation in base composition, repetitive sequences, or mutational processes that genome-wide randomization does not capture. Consistent with this, G-run density was elevated around G4 motifs in low-GC genomes with high ratios under the analytical expectation (2.3-fold the genome mean within 200 bp of the motif and 1.8-fold at 800–1,000 bp, compared with 1.1 and 1.0 in high-GC genomes; Supplementary Figure S6b), and when expected counts were recomputed from randomizations that preserve composition within 1-kb windows, only 16 of the 83 significantly enriched genomes remained enriched (Supplementary Figure S6c). Repeated sequences also contributed in some genomes (Supplementary Figure S6d). We therefore interpret obs/exp ratios as enrichment relative to the null model rather than as direct evidence of evolutionary selection.

Several species showed exceptionally high obs/exp ratios (Figure 3d). *Bartonella schoenbuchensis*, an intracellular pathogen of ruminants, exhibited the highest obs/exp ratio in the entire dataset: 80-fold enrichment at only 38.0% GC, harboring 187 G4 motifs where randomized genomes predict about two; however, 76 of these motifs repeat a sequence found elsewhere in the genome, and the excess disappeared when local composition was preserved. *Helicobacter pylori*, the causative agent of gastric ulcers, showed 8-fold enrichment (41 G4s at 38.5% GC). *Campylobacter sputorum* exhibited 18-fold enrichment with only 2 G4s at 29.7% GC, a ratio driven largely by the near-zero expected count at such low GC content. *Capnocytophaga leadbetteri* and *C. sputigena* showed 13–16-fold enrichment (GC = 39–40%), and *Prevotella melaninogenica* and *P. scopos* showed 11–19-fold enrichment (GC = 41%). Notably, the majority of the genomes with the highest ratios belong to recognized human pathogens or opportunistic pathogens associated with periodontal disease or other infections (Figure 3d). Of these, *P. melaninogenica*, *P. scopos*, *C. leadbetteri*, *C. sputigena* and *Helicobacter pylori* remained enriched after controlling for local composition (Supplementary Figure S6c).

We further investigated whether the functional profile of promoter G4 genes differs between high obs/exp genomes and genomes with expected obs/exp ratios (Figure 3c). Comparing genomes with obs/exp >3 against those with obs/exp between 0.7 and 1.5, we found that mobile genetic element genes were 3.2-fold enriched among promoter G4 genes in outlier genomes (FDR q < 0.001), while the 1.4-fold excess of virulence-associated genes was not significant. Conversely, transcription and transport genes were proportionally less abundant in outlier genomes (0.4- and 0.7-fold, q < 0.05). This functional shift indicates that, in the genomes with the highest G4 enrichment, promoter G4 motifs are more often located upstream of genes involved in genome plasticity than upstream of transcription and transport genes.

### 2.4 Promoter G4 motifs are rarely shared among congeneric species

To examine whether promoter G4 motifs are shared among related species, we analyzed cross-species conservation within 35 genera containing three or more species in our dataset. For each genus, we identified all orthogroups bearing a promoter G4 in at least one species and computed a conservation score defined as the fraction of species within the genus in which that orthogroup had a promoter G4 (Figure 4). Orthogroups were inferred with OrthoFinder (Emms and Kelly, 2019) (Supplementary Table S4; Supplementary Methods S2). Because G4 presence was scored independently in each species, this score indicates whether a promoter G4 occurs upstream of the same orthologous gene; conservation of the G4-forming sequence itself was assessed separately by aligning orthologous promoters (see below).

**FIGURE 4:**
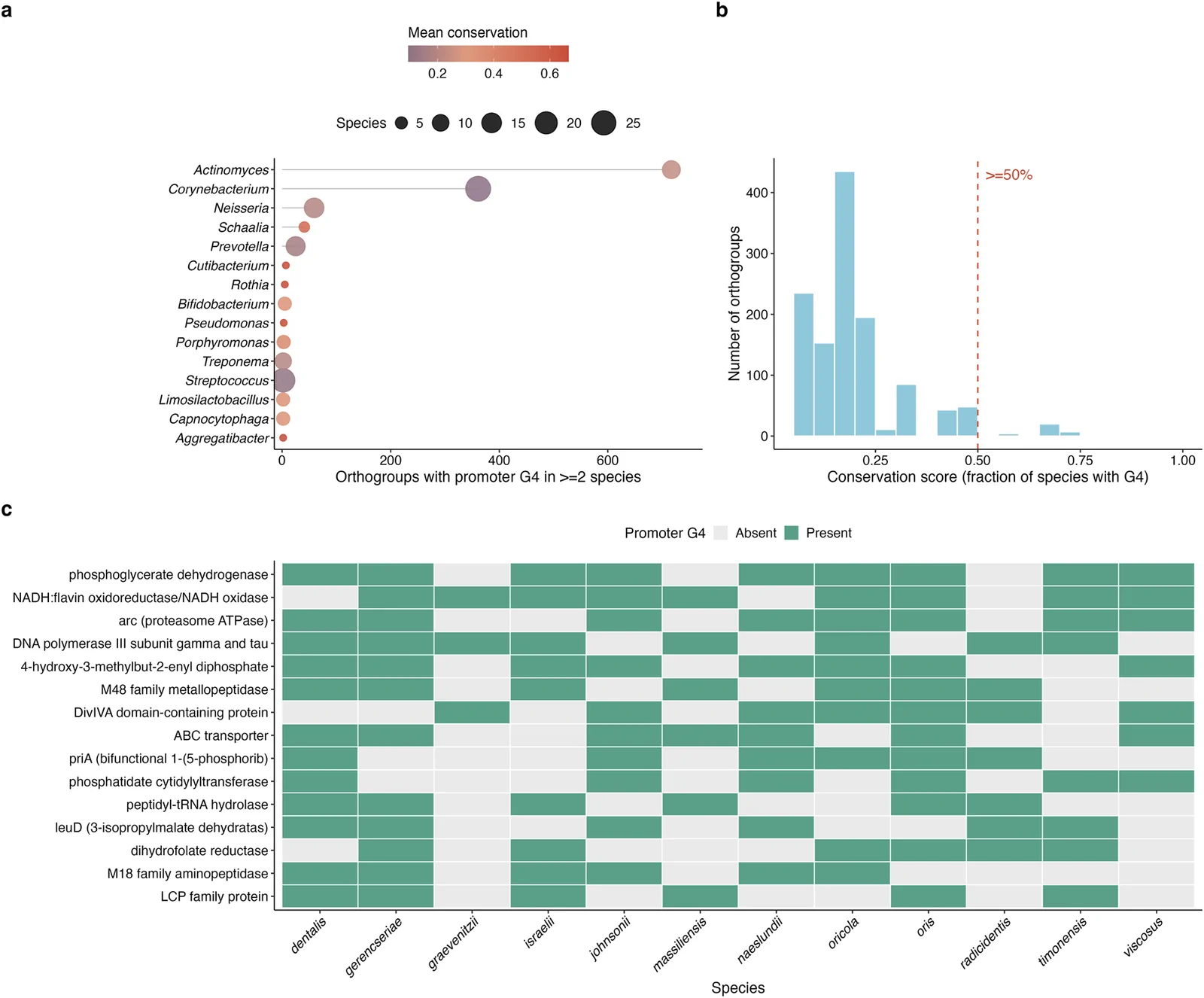
Cross-species conservation of promoter G4 occurrence within genera. **(a)** Per-genus summary of cross-species G4 conservation for genera with three or more species and at least one shared promoter G4 orthogroup. Point size indicates the number of species in the genus; color represents the mean conservation score. *Actinomyces* and Corynebacterium show the largest numbers of orthogroups with promoter G4s shared across multiple species. **(b)** Distribution of conservation scores (fraction of species with a promoter G4 at a given orthogroup) across all 1,239 orthogroup–genus pairs. The dashed red line marks the 50% threshold; 79 orthogroups reach this threshold. **(c)** Orthogroup-by-species presence/absence matrix for the 15 orthogroups with the most widely shared promoter G4s in *Actinomyces* (12 species). Green tiles indicate presence of a promoter G4; grey tiles indicate absence. These orthogroups have promoter G4s in 6–9 of 12 species.

The analysis yielded 1,239 orthogroup–genus pairs in which a promoter G4 was shared across at least two species of the same genus. The distribution of conservation scores was strongly left-skewed (Figure 4b): 65.1% of orthogroups had conservation scores below 0.2, meaning the promoter G4 was present in fewer than 20% of congeneric species. Only 79 orthogroup–genus pairs reached a conservation score of 0.5 (majority-conserved), and only one, a BolA family protein in *Kingella* (3 of 3 species), carried a promoter G4 in all species of a genus. Among larger genera, the maximum was 0.75 (9 of 12 species), for an NADH:flavin oxidoreductase and phosphoglycerate dehydrogenase in *Actinomyces*.

*Actinomyces* (12 species) and *Corynebacterium* (27 species) showed the largest numbers of orthogroups with shared promoter G4s (Figure 4a). In *Actinomyces*, 17 orthogroups had promoter G4s in a majority of species (Figure 4c), including NADH:flavin oxidoreductase and phosphoglycerate dehydrogenase (9/12 species), *arc* (proteasome-associated ATPase), 4-hydroxy-3-methylbut-2-enyl diphosphate reductase and DNA polymerase III subunit γ/τ (8/12), and *trpA*, *priA* and *leuD* (6/12). For *arc*, the G4 was at the same aligned position in 56 of 80 alignable comparisons of orthologous promoters. These conserved genes encode core metabolic enzymes involved in amino acid biosynthesis (*trpA*, *leuD*), serine and isoprenoid biosynthesis, purine salvage (*priA*), and protein quality control (*arc*), which makes these promoters candidates for experimental tests of G4-mediated regulation.

*Corynebacterium*, despite having the second-largest number of orthogroups with shared promoter G4s (361), had none conserved in a majority of species. This pattern of widespread but shallow conservation reflects the depth of the genus: species-tree distances within *Corynebacterium* reached 1.6 substitutions per site, about three times the maximum in *Neisseria*, and only 10.3% of comparisons between orthologous promoters could be aligned. Across genera, the proportion of G4 motifs retained at the same aligned position declined with phylogenetic distance in 7 of 9 genera with sufficient data (combined permutation p = 3 × 10^−9^; Supplementary Figure S7b), and where a G4 was absent from an alignable orthologous promoter, the aligned sequence was 1 G substitution away from a canonical motif in 19.4% of cases, compared with 1.6% in unrelated promoters (Supplementary Figure S7b). Other genera such as *Neisseria* (15 species), *Prevotella* (14 species), and *Streptococcus* (23 species) showed fewer shared promoter G4 orthogroups (59, 25 and 2, respectively), potentially reflecting greater ecological divergence among congeneric species or, in the case of low-GC genera like *Streptococcus* (mean GC = 39%), an overall paucity of G4 motifs.

Beyond the genome-wide distribution, this comparison shows that the set of genes carrying promoter G4 motifs differs substantially even among species of the same genus, and that a promoter G4 at the same gene is present in most species of a genus in only a few cases, most notably in *Actinomyces*. Sequence-level comparison of orthologous promoters showed that promoter G4 motifs were shared among congeneric species more often than expected by chance (3,461 species pairs observed versus 1,922 expected), but their G-runs were kept intact no more often than other GGG runs in the same promoters (43.1% versus 42.8%; Supplementary Figure S7c). G4 motifs in genomes enriched relative to randomized genomes (Section 2.3) were not retained more often than those of other genomes in the same genus; they were retained less often (−12 percentage points, permutation p = 0.0006; Supplementary Figure S7d).

## 3 Discussion

This study presents the first community-wide survey of G-quadruplex motifs in a defined microbial community, covering 381 genomes from 150 genera of oral bacteria. G4 motifs were enriched in promoter regions, occurred above the level expected from sequence composition in a small subset of genomes, mainly oral Bacteroidota, Campylobacterota and Pseudomonadota, and were preferentially associated with virulence and environmental response genes. These are genomic associations and do not by themselves demonstrate regulatory roles.

Several of these observations extend earlier genome-wide surveys of bacterial G4s rather than being entirely new. Previous studies documented G4-forming sequences in individual bacterial genomes, including *E. coli*, where G4 motifs are enriched in regulatory regions (Perrone et al., 2017; Kota et al., 2015; Cahoon and Seifert, 2009; Rawal et al., 2006). Larger surveys have since catalogued G4-forming sequences across many bacterial genomes (Yadav et al., 2007; Bartas et al., 2019), and a recent analysis of 1,566 bacterial genomes showed that their distribution, including enrichment near gene starts, cannot be explained by GC content or genome size alone (Fu et al., 2025a). That study found fewer G4 motifs than in randomized sequences above ∼55% GC; our randomized genomes preserving base composition reproduce this depletion, whereas with dinucleotide-preserving randomized genomes high-GC oral genomes were close to expectation. Relative to these studies, the present work covers a defined, clinically relevant community, links promoter G4 motifs to the functions of their downstream genes, identifies genomes whose G4 counts exceed expectation even when local composition is preserved, and compares G4 motifs between aligned orthologous promoters. Alternative nucleic acid structures, including G4s, are increasingly recognized as regulators of microbial gene expression and as potential antimicrobial targets (Yousuf and Bhat, 2026); the associations reported here identify candidate loci for testing such roles experimentally.

Our results also show how strongly conclusions about G4 enrichment depend on the null model: the over-representation of G4 motifs in low-GC genomes suggested by a simple analytical expectation disappeared with dinucleotide-preserving randomized genomes, and most of the remaining excess was shared with the surrounding sequence. G-rich regions, local compositional heterogeneity and repeats can therefore produce apparent enrichment without any constraint on G4 motifs themselves, so we interpret obs/exp ratios as enrichment relative to the stated null model rather than as evidence of selection.

The genomes that remain enriched after controlling for local composition are the most promising candidates for G4-specific functions, and several belong to organisms with documented roles in human disease, including *P. melaninogenica*, *Capnocytophaga* species, *Kingella kingae* and *H. pylori*, in which G4-forming sequences have been proposed to contribute to genetic plasticity; *Neisseria* species, of which *N. sicca* remained enriched here, use G4 structures for pilin antigenic variation (Cahoon and Seifert, 2013).

The consistent 2.5–3-fold enrichment of G4 motifs in promoter regions across phylogenetically diverse genera shows that G4 motifs are not randomly distributed with respect to genes, but positional enrichment alone does not establish a role in transcription initiation, because G4 motifs near gene starts could also arise from sequence features unrelated to G4 formation. Experimental studies have shown that G4s in bacterial promoter or upstream regions can modulate gene expression (Perrone et al., 2017; Kota et al., 2015; Holder and Hartig, 2014), and G4 folding at promoters is a well-characterized mechanism of transcriptional control in eukaryotes, for example, at the human c-MYC oncogene (Siddiqui-Jain et al., 2002). In the oral cavity, where bacteria experience rapid fluctuations in pH, nutrient availability, oxygen tension, and immune challenge (Hajishengallis and Lamont, 2012), G4-based regulation could in principle provide rapid, reversible control of gene expression; whether the motifs identified here fold and affect transcription remains to be tested.

Comparison of orthologous promoters showed that promoter G4 motifs are inherited within genera but turn over rapidly: they were shared among congeneric species more often than expected by chance, sharing declined steeply with phylogenetic distance, and many absences involved a single G substitution. The G-runs of G4 motifs were not more conserved than other G-runs in the same promoters, and G4 motifs in enriched genomes were, if anything, retained less often than those of their congeners, so we found no evidence that the G4-forming sequence is maintained by purifying selection over the divergence times sampled. Unlike deeply conserved core promoter elements, promoter G4 motifs thus behave as inherited but evolutionarily labile features, as in eukaryotic regulatory regions (Huppert and Balasubramanian, 2005; Huppert and Balasubramanian, 2007). The few orthologous promoters that retain a G4 in most species of a genus, such as *arc* in *Actinomyces*, are the best candidates for conserved G4-associated regulation.

Several limitations should be acknowledged. First, G4 formation *in vivo* depends on factors not modeled here, including supercoiling, K^+^ concentration, molecular crowding and G4-resolving helicases (Mendoza et al., 2016; Brosh, 2013), and would need to be confirmed with approaches such as G4-seq (Chambers et al., 2015), BG4 immunoprecipitation (Biffi et al., 2013), or G4-stabilizing ligands (Neidle, 2016). Second, orthologous promoters of distant congeners often could not be aligned, and draft assemblies may hide some orthologs, so retention could be assessed reliably mainly between close relatives. Third, keyword-based functional classification captures only a subset of gene functions. Fourth, only canonical three-tetrad motifs were considered. Fifth, randomized genomes preserve composition only up to a given order and spatial scale, and the choice of null model changed the apparent dependence of enrichment on GC content (Supplementary Figure S6a). Future work should test G4 formation at the predicted sites, particularly in the genomes that remain enriched after controlling for local composition, and explore G4-stabilizing small molecules (Neidle, 2016) as modulators of bacterial gene expression and antimicrobial agents (Yousuf and Bhat, 2026); extending this framework to other microbial communities would show whether these patterns are specific to the oral microbiome.

## 4 Conclusion

Our systematic survey of 48,234 G-quadruplex motifs across 381 oral bacterial genomes establishes the first comprehensive atlas of G4 distribution in a defined microbial community. Three principal findings emerge: (i) G4 motifs are 2.5–3-fold enriched in promoter regions relative to their genomic proportion, a positional association that may reflect roles in transcription; (ii) after accounting for dinucleotide composition, G4 motifs are not systematically over-represented in low-GC genomes, and only a small set of genomes, mainly oral *Prevotella*, *Capnocytophaga*, *Kingella* and *Neisseria* species and *H. pylori*, carry more G4 motifs than expected from their local sequence composition; and (iii) promoter G4s are preferentially associated with virulence, transport, and regulatory genes, and are inherited within genera but lost rapidly as species diverge, with a few orthologous promoters, such as *arc* in *Actinomyces*, retaining a G4 in most species. If G4 formation and function at these loci are confirmed experimentally, G4 structures could become targets for antimicrobial strategies, for example, G4-stabilizing small molecules that modulate virulence gene expression in oral pathogens.

## 5 Materials and methods

### 5.1 Genome dataset

A total of 381 NCBI RefSeq reference genomes representing 150 genera of oral bacteria were obtained from the NCBI RefSeq database. Each genome was accompanied by a paired GFF3 annotation file. Genomes spanned a GC content range of 23.3%–74.4% and sizes from 0.81 to 9.52 Mb. Genome sequences were stored as compressed FASTA files organized by genus and species. Species were selected according to the Human Oral Microbiome Database (HOMD), and one NCBI RefSeq reference genome was retained per species.

### 5.2 G4 motif prediction

G-quadruplex motifs were predicted using g4predict (Parker, 2026) with the following parameters: minimum number of G-quartet stacks (tetrads) = 3, maximum loop length = 7 nucleotides, no bulges permitted. Predictions were performed on both strands of each decompressed genome FASTA file. The output was generated in BED12 format, in which blocks represent G-runs and structural features are encoded in the name field using the format {tetrads}t{bulges}b{loop1},{loop2},{loop3}l. Both BED6 and BED12 outputs were retained for downstream analysis.

### 5.3 Genomic region annotation

Each G4 motif was assigned to exactly one genomic region using a priority-based classification hierarchy to prevent double-counting: promoter (defined as 300 bp upstream of the nearest CDS start site) > rRNA > tRNA > CDS > intergenic. Gene annotations were parsed from GFF3 files using gffutils (version 0.13). Intersections between G4 coordinates and genomic regions were computed using pybedtools (Dale et al., 2011), a Python wrapper for BEDTools (Quinlan and Hall, 2010). For each G4 overlapping multiple regions, the region with the highest priority was assigned. The nearest gene and its product annotation were recorded for each G4 motif.

### 5.4 Functional enrichment analysis

Gene products from GFF3 annotations were classified into 11 functional categories based on keyword matching against a curated dictionary: metabolism, transport, transcription, translation, replication, DNA repair, cell wall, virulence, stress response, signal transduction, and mobile genetic elements. Enrichment of each category among genes with at least one promoter G4 motif versus all annotated genes was assessed using Fisher’s exact test. Multiple testing correction was performed using the Benjamini–Hochberg procedure to control the false discovery rate at 5%. Enrichment ratios were computed as (G4_genes_in_category/total_G4_genes)/(all_genes_in_category/total_genes).

### 5.5 Observed-to-expected ratio analysis

For each genome, expected G4 counts were obtained by predicting G4 motifs, with the same g4predict settings, in randomized genomes that preserve its dinucleotide composition (first-order Markov chains fitted to each contig; 10–50 randomized genomes per genome). Observed and expected counts were compared with one-sided Poisson tests and Benjamini–Hochberg correction across the 381 genomes, and log_2_ ((observed +0.5)/(expected +0.5)) was used as the effect size. Randomized genomes preserving base or tetranucleotide composition, or composition within 1-kb and 200-bp windows, were used as sensitivity analyses and to test the contribution of local composition. Residual variation in ln (G4 density) after GC content was analysed by adding phylum and genome size to a linear model. Details of the null models and of the flanking-sequence, repeat and residual analyses are given in Supplementary Methods S1.

### 5.6 Cross-species conservation analysis

For the 35 genera with three or more species (242 genomes), orthogroups were inferred separately for each genus with OrthoFinder 2.5.5^28^, and single-copy orthologs were used. The promoter region of each gene carrying a promoter G4 was aligned with that of its ortholog in every congeneric species; the G4 was scored as retained when a predicted motif occupied the aligned position, and as a near-G4 when one substitution would restore a canonical motif. Retention was related to phylogenetic distance using species trees built from 150 single-copy orthogroups, compared with that of other G-runs in the same promoters, and tested against chance sharing by permutation. Full details are given in Supplementary Methods S2.

### 5.7 G4 structural characterization

G4 structural features were extracted from g4predict BED12 output files. The number of tetrads and loop lengths were parsed from the BED12 name field. G-run lengths were derived from BED12 block sizes. Mean loop length was computed for each motif. Loop symmetry was classified based on whether all three loops had equal length. Loop class annotations (short: 1–2 nt, medium: 3–5 nt, long: 6–7 nt) were assigned to each individual loop.

### 5.8 Strand bias analysis

For each genome, G4 motifs were classified as template or non-template strand relative to the gene whose promoter or coding sequence contained the motif, using the annotated strand of that gene: motifs on the same strand as the gene were classified as non-template and motifs on the opposite strand as template. Intergenic motifs and motifs assigned to genes on both strands were excluded. A bias ratio was computed as the fraction of G4 motifs on the template strand. Statistical significance of strand bias was assessed using a two-tailed binomial test (null: 50% on each strand). Results were aggregated at the genus level by summing template and non-template counts across all species within each genus. Genera with at least 10 genic G4 motifs were tested, and p-values were corrected with the Benjamini–Hochberg method.

## Data Availability

The datasets presented in this study can be found in online repositories. The names of the repository/repositories and accession number(s) can be found in the article/Supplementary Material.
