# Supplementary material for: Genome-wide distribution and putative regulatory roles of G-quadruplex motifs in oral bacteria
Source: Front Genet. 2026 Oct 29;17:1961845. doi: 10.3389/fgene.2026.1961845 (PMC13754242; doi:10.3389/fgene.2026.1961845)
Supplement: Supplementary file 1 [file Supplementaryfile1.pdf]

Supplementary Information

Genome-Wide Distribution and Putative Regulatory Roles of G-Quadruplex Motifs in Oral Bacteria

Yizhou Wang[1,*], Zongyun Fu[1], Pengcheng Gao[1], Yaozhong Chen[1]

[1] Department of Operative Dentistry and Endodontics, Zhongda Hospital, Medical College, Southeast University, Nanjing, China.

* Correspondence to: Yizhou Wang (756991473@qq.com)

Supplementary Methods

Supplementary Methods S1. Null models, flanking-sequence, repeat and residual analyses. For each genome, randomized genomes were generated contig by contig with a custom C program under six null models: an exact shuffle of bases (base composition); first- and third-order Markov chains fitted to each contig (preserving dinucleotide and tetranucleotide composition, respectively); exact base shuffles within consecutive 1-kb and 200-bp windows; and a first-order Markov chain fitted within each 1-kb window. Non-ACGT characters were kept in place. G4 motifs were predicted in each randomized genome with the same g4predict command as for the real genomes. The expected count was the mean over at least 10 randomized genomes, extended to up to 50 when fewer than 30 motifs had accumulated, with 0.5 added to the summed count before averaging. Enrichment and depletion were tested with one-sided Poisson tests of the observed count against the expected count, with Benjamini–Hochberg correction across the 381 genomes; $\log_2$((observed + 0.5)/(expected + 0.5)) was used as the effect size. The dinucleotide-preserving model was used for the main analysis and the other models as sensitivity analyses. Differences between GC quartiles were assessed with the Mann–Whitney U test. Promoter-G4 gene categories were compared between genomes with obs/exp > 3 and genomes with obs/exp of 0.7–1.5 (Fig. 3c) with Fisher's exact tests and Benjamini–Hochberg correction. Around each G4 motif, the density of G-runs (three or more consecutive G or C) was measured in 50-bp bins up to 1 kb on each side and expressed relative to the genome mean, and motifs whose exact sequence occurred more than once in a genome were counted as repeated. For the residual analysis, ln(G4 density) of genomes with at least one G4 motif was regressed on GC content, and phylum (phyla with at least five genomes, from the NCBI lineage; others pooled) and log genome size were added and tested with F-tests. Obs/exp ratios quantify enrichment relative to the stated null model and were not interpreted as measures of selection.

Supplementary Methods S2. Orthology-based analysis of promoter G4 conservation. Cross-species comparison was performed for the 35 genera containing three or more species in the dataset (242 genomes). Protein sequences were taken from the RefSeq annotation of each genome (one per protein-coding locus), and orthogroups were inferred separately for each genus with OrthoFinder version 2.5.5 [1] using DIAMOND [2] with default settings. Only genes present as a single copy in both genomes of a comparison were treated as orthologs. For each gene, the region from 300 bp upstream to 30 bp downstream of the start codon was extracted in gene orientation, and the G4 motifs predicted above that overlapped the 300-bp upstream region were recorded; motifs extending beyond the extracted region (about 5%) were excluded. For each promoter G4 and each congeneric species with a single-copy ortholog, the two promoter regions were aligned by global pairwise alignment with affine gap penalties (Biopython PairwiseAligner [3]). A comparison was considered alignable when sequence identity over the 50 nt on each side of the motif (motif excluded) exceeded the 95th percentile of the same measure obtained by aligning the promoter with randomly chosen, non-orthologous promoters of the same congeneric species. In alignable comparisons, the G4 was scored as retained when a predicted G4 motif overlapped the aligned position; otherwise the aligned sequence (±10 nt) was scanned for the minimum number of substitutions needed to form a canonical motif (four runs of three guanines separated by loops of 1–7 nt), and sites requiring one substitution were classified as near-G4. The same scan applied to aligned positions in unrelated promoters provided the background rate. Conservation of the G4-forming sequence was assessed by comparing, within each alignment, the fraction of the four G-runs kept intact with that of other GGG runs on the same strand, both within 50 nt of the motif and across the whole promoter, using paired Wilcoxon signed-rank tests on per-site means. Species trees with branch lengths were built for each genus from a concatenated alignment of 150 randomly selected single-copy orthogroups (MAFFT [4]; FastTree with the LG model and gamma-distributed rates [5]; midpoint rooting), and the relationship between the proportion of G4 motifs retained and patristic distance was tested within each genus by

permuting species labels (4,999 permutations; one-sided), with p-values combined by Fisher's method. To test whether promoter G4 motifs are shared more often than expected by chance, promoter G4 status was permuted among the single-copy genes of each species (999 permutations), keeping the number of G4-bearing genes per species, and the number of species pairs sharing a promoter G4 at the same orthogroup was compared with the permuted distribution. Retention in genomes significantly enriched relative to dinucleotide-preserving randomized genomes (section 5.5) was compared with that in other genomes of the same genus as a weighted mean of within-genus differences, with labels permuted within genera (9,999 permutations; two-sided). The orthogroup-level conservation score in Supplementary Table S4 is the number of species with a promoter G4 divided by the number of species in the genus, as in Fig. 4; the fraction of species carrying a single-copy ortholog that have a promoter G4 is given in an additional column.

Supplementary Tables

Supplementary Table S1. Complete genome summary statistics. Summary statistics for all 381 genomes, including genus, species, accession, genome size, GC content, G4 count, density, genomic context distribution, and the expected G4 count, obs/exp ratio, FDR-adjusted Poisson p-values and resulting classification relative to dinucleotide-preserving randomized genomes. (Table_S1_genome_summary_v6.xlsx)

Supplementary Table S2. Complete G4 motif catalog. Coordinates, strand, score, genomic region, nearest gene, and product annotation for all 48,234 predicted G4 motifs. (all_g4_annotated.xlsx)

Supplementary Table S3. Functional enrichment results. Fisher's exact test results for 11 functional categories among promoter-G4 genes, with enrichment ratios and Benjamini–Hochberg adjusted p-values. (functional_enrichment.xlsx)

Supplementary Table S4. Promoter G4 conservation across orthologous genes. For every OrthoFinder orthogroup in the 35 genera with three or more species that carries a promoter G4 in at least one species: number of species in the genus, number carrying a single-copy ortholog, number with a promoter G4, conservation score (species with a promoter G4 divided by species in the genus), fraction of ortholog-carrying species with a promoter G4, list of species with a promoter G4, gene name and product, and the numbers of alignable pairwise comparisons and of comparisons in which the G4 was at the same aligned position. (Table_S4_orthogroup_conservation_v6.xlsx)

Supplementary Table S5. Observed and expected G4 counts under randomized-genome **null models.** For all 381 genomes: phylum, GC content, observed G4 count and, for each of six null models (base, dinucleotide and tetranucleotide composition of the whole genome; base composition within 1-kb and 200-bp windows; dinucleotide composition within 1-kb windows), the number of randomized genomes, expected count, observed/expected ratio, Benjamini–Hochberg-adjusted Poisson p-values for enrichment and depletion and the resulting classification, together with the fraction of G4 motifs whose sequence occurs more than once in the genome. (Table_S5_obsexp_shuffled_nulls.xlsx)

Supplementary Figures

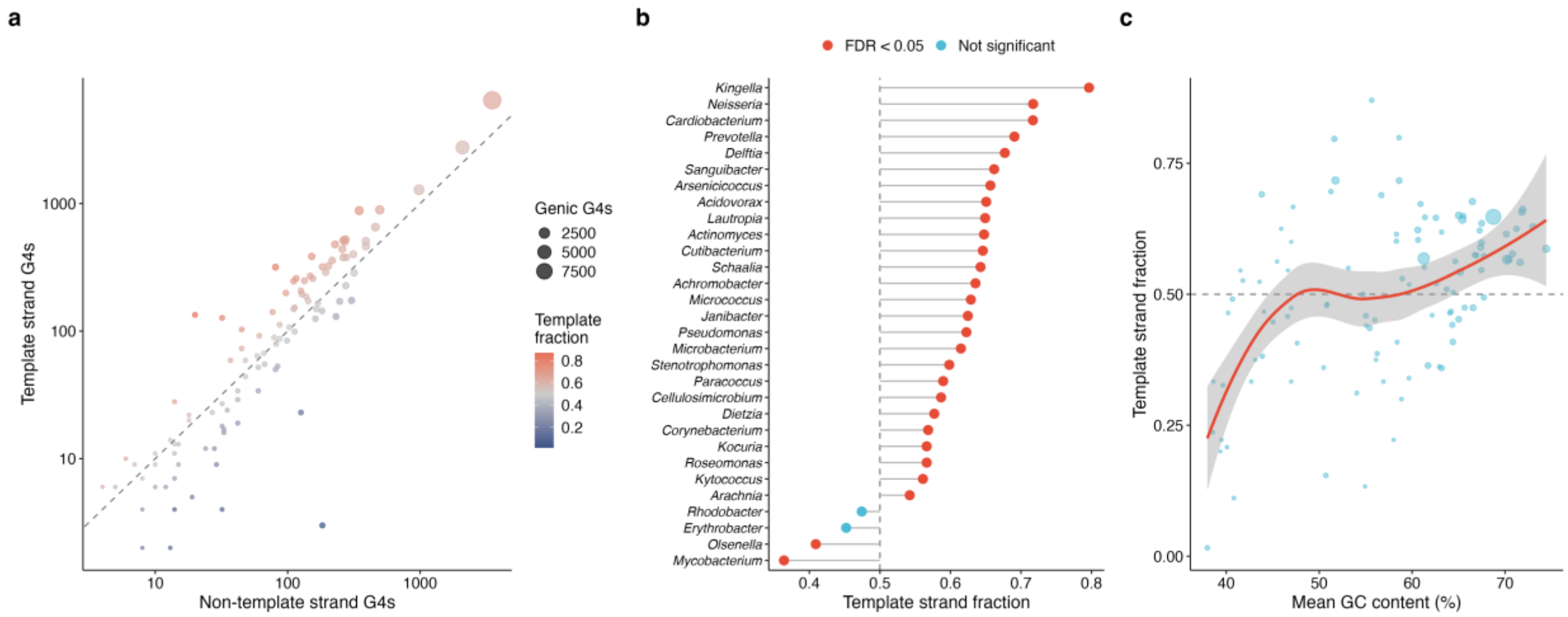

Supplementary Figure S1. Strand bias analysis of G4 motifs. (a) Numbers of G4 motifs on the template and non-template strands per genus (log scales), for the 106 genera with at least 10 G4 motifs in promoters or coding sequences; strand is assigned relative to the gene containing the motif. Colour shows the template strand fraction and point size the number of genic G4 motifs. (b) Template strand fraction for the 30 genera with the most genic G4 motifs; red points indicate significant bias (FDR < 0.05, binomial test). (c) Template strand fraction vs. mean GC content per genus, with LOESS regression and its 95% confidence band.

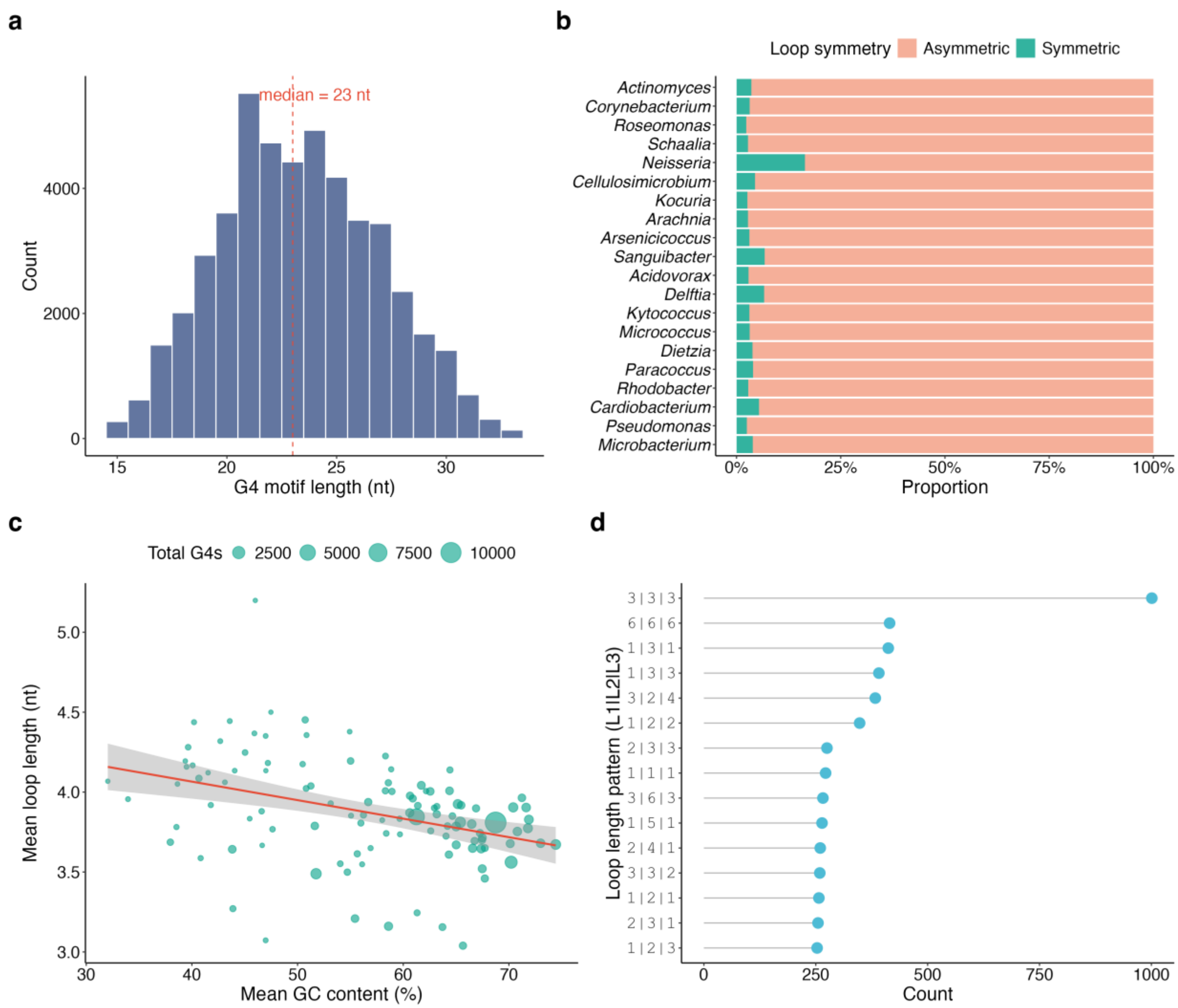

Supplementary Figure S2. G4 structural features. (a) G4 motif length distribution (median = 23 nt). (b) Loop symmetry by genus (top 20). (c) Mean loop length vs. GC content across genera. (d) Top 15 loop length patterns.

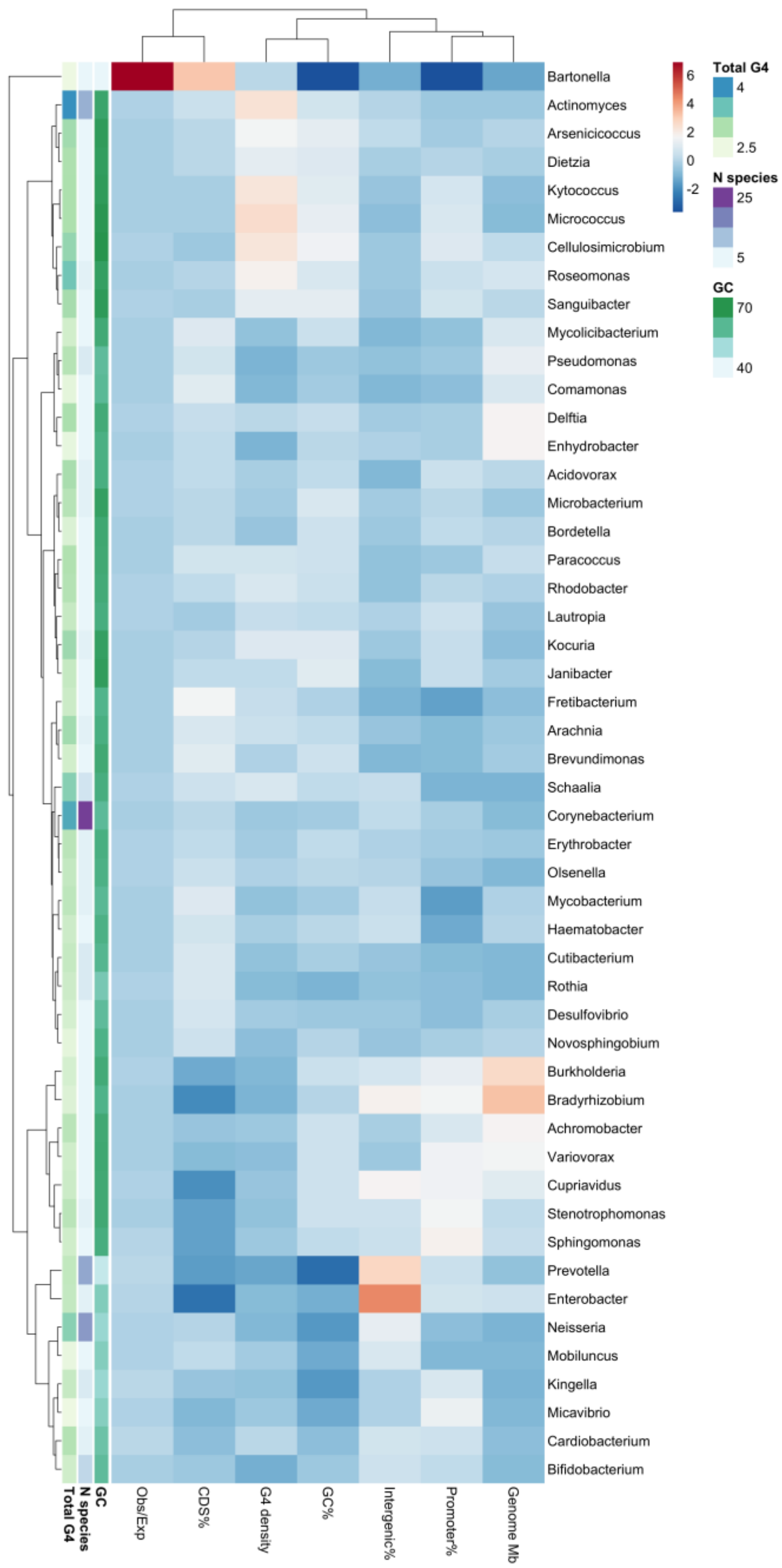

Supplementary Figure S3. Extended genus-level heatmap. Hierarchical clustering heatmap of z-scored G4 features (observed/expected ratio relative to dinucleotide-preserving randomized

genomes) for the top 50 genera, with row annotations for GC content, species count, and total G4 count.

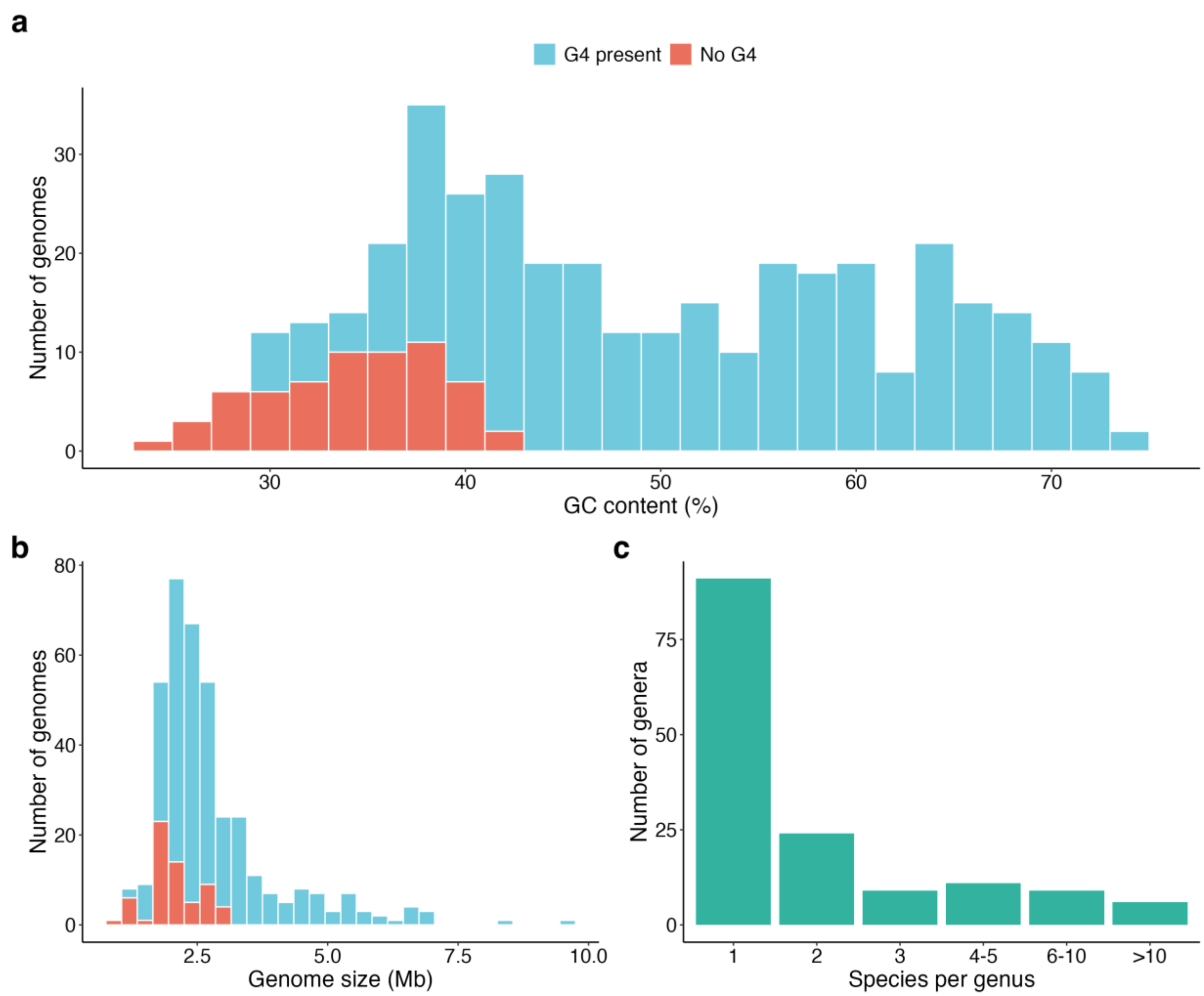

Supplementary Figure S4. Dataset overview. (a) GC content distribution colored by G4 presence/absence. (b) Genome size distribution. (c) Species per genus distribution.

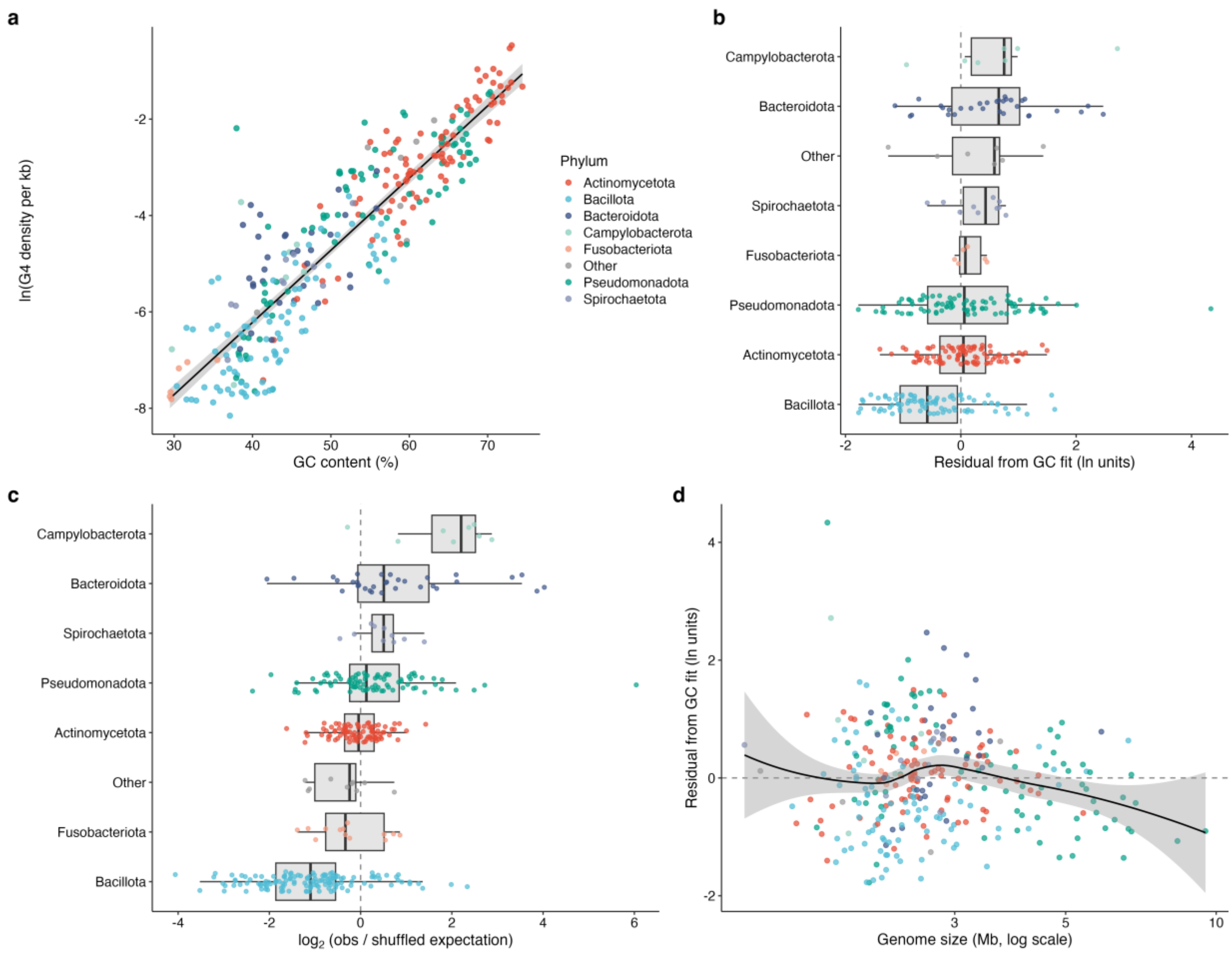

Supplementary Figure S5. Variation in G4 density not explained by GC content. (a) G4 density against GC content for the 318 genomes with at least one G4 motif, coloured by phylum, with the least-squares fit. (b) Residuals from the GC fit by phylum. (c) Log_2 ratio of observed to expected G4 counts by phylum for all 381 genomes, with expected counts from dinucleotide-preserving randomized genomes. (d) Residuals from the GC fit against genome size, with a loess fit.

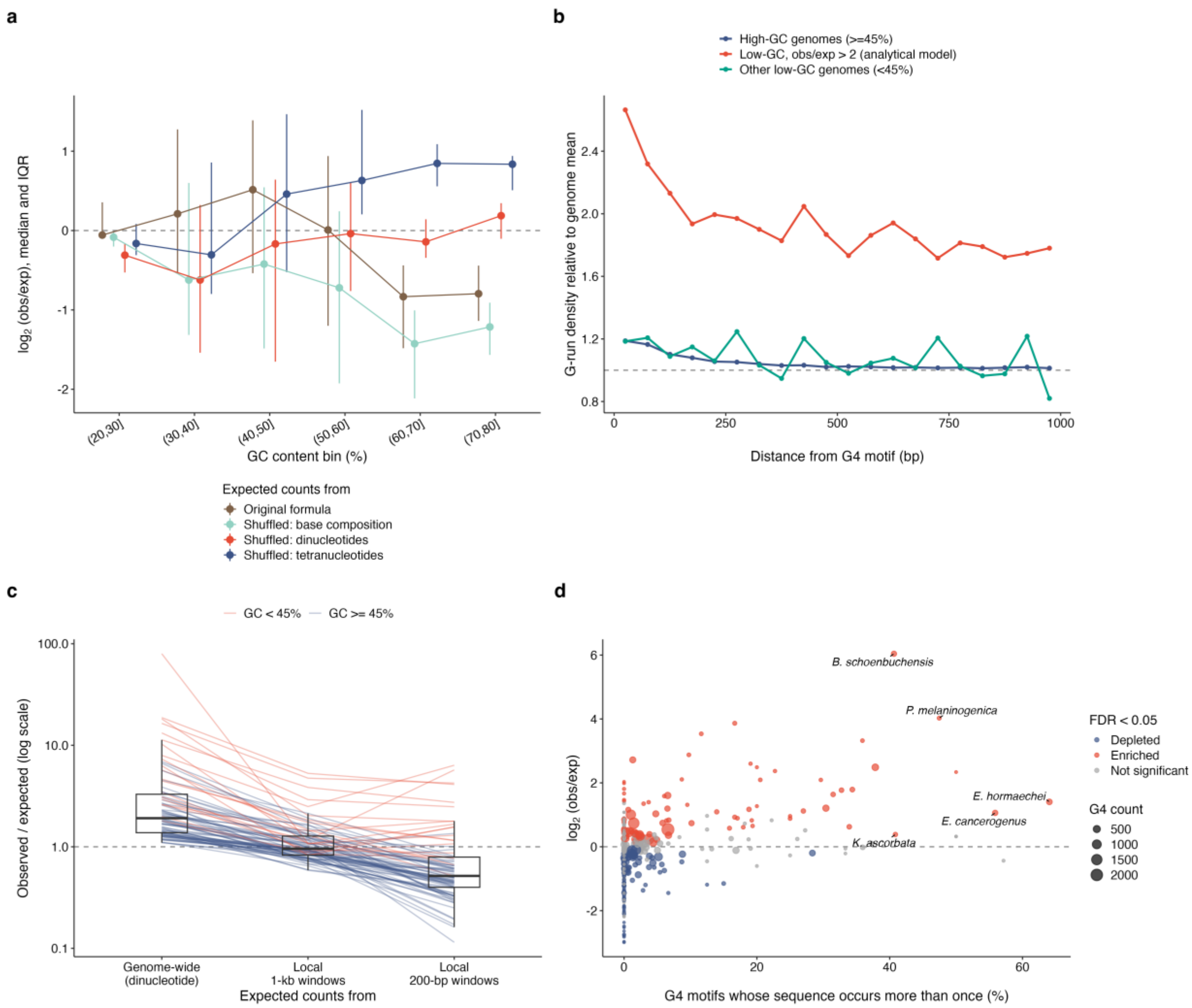

Supplementary Figure S6. Null models, local composition and repeats. (a) Median and interquartile range of the $\log_2$ ratio of observed to expected G4 counts in 10% GC bins, with expected counts from the analytical expectation $E(G4) = L \times (GC/2)^{12}$ or from randomized genomes preserving base, dinucleotide or tetranucleotide composition. (b) Density of G-runs (three or more consecutive G or C) around G4 motifs relative to the genome mean, in 50-bp bins up to 1 kb from the motif (mean over motifs in genomes with at least five G4 motifs), for low-GC genomes with obs/exp > 2 under the analytical expectation, other genomes below 45% GC and genomes of 45% GC or more. (c) Observed/expected ratios of the 83 genomes significantly enriched against genome-wide dinucleotide-preserving randomization, recomputed with

randomizations that preserve composition within 1-kb or 200-bp windows; lines join the same genome. (d) $\log_2$ ratio of observed to expected counts against the percentage of G4 motifs whose exact sequence occurs more than once in the genome.

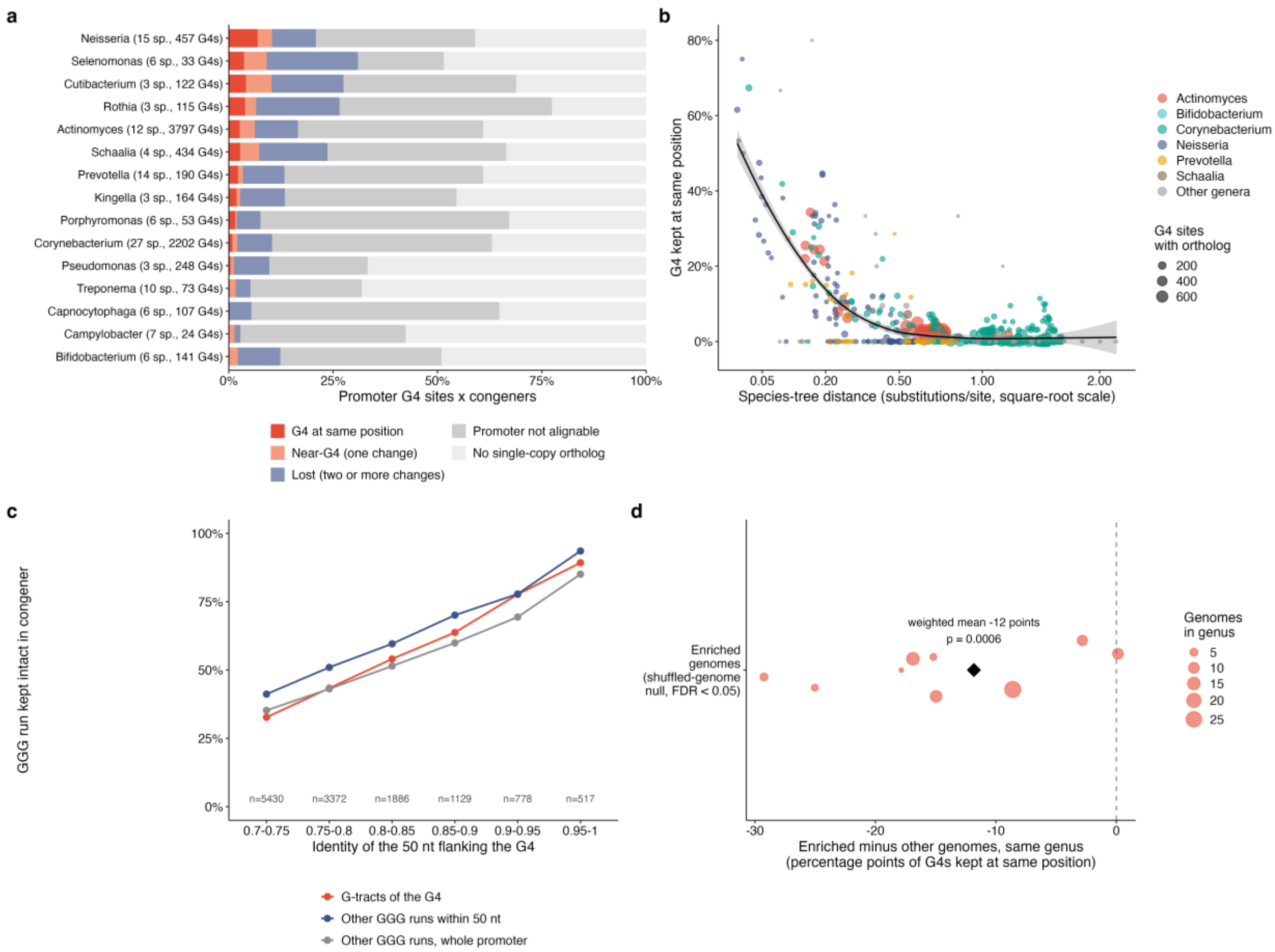

Supplementary Figure S7. Sequence-level comparison of orthologous promoters. (a) Fate of each promoter G4 motif in every congeneric species, for genera with at least 20 promoter G4 motifs among the 35 genera with three or more species: G4 at the same aligned position, near-G4 (one G substitution from a canonical motif), lost (two or more substitutions), orthologous promoter not alignable, or no single-copy ortholog. Genera are ordered by the proportion retained. (b) Proportion of promoter G4 motifs retained at the same position against species-tree distance, one point per species pair (both directions pooled); point size shows the number of G4 motifs with a single-copy ortholog, and the line is a loess fit. (c) Proportion of G-runs kept intact in the

congeneric species for the four G-runs of G4 motifs, other GGG runs within 50 nt of the motif, and other GGG runs in the whole promoter, binned by identity of the flanking sequence; n, number of alignable comparisons per bin. (d) Difference in the proportion of G4 motifs retained between genomes significantly enriched relative to dinucleotide-preserving randomized genomes (FDR < 0.05) and other genomes of the same genus, one point per genus (point size, number of genomes); the diamond shows the weighted mean with its permutation p-value.

Supplementary References

1. Emms, D. M. & Kelly, S. OrthoFinder: phylogenetic orthology inference for comparative genomics. *Genome Biol.* **20**, 238 (2019).

2. Buchfink, B., Reuter, K. & Drost, H.-G. Sensitive protein alignments at tree-of-life scale using DIAMOND. *Nat. Methods* **18**, 366–368 (2021).

3. Cock, P. J. A. et al. Biopython: freely available Python tools for computational molecular biology and bioinformatics. *Bioinformatics* **25**, 1422–1423 (2009).

4. Katoh, K. & Standley, D. M. MAFFT multiple sequence alignment software version 7: improvements in performance and usability. *Mol. Biol. Evol.* **30**, 772–780 (2013).

5. Price, M. N., Dehal, P. S. & Arkin, A. P. FastTree 2 – approximately maximum-likelihood trees for large alignments. *PLoS ONE* **5**, e9490 (2010).
